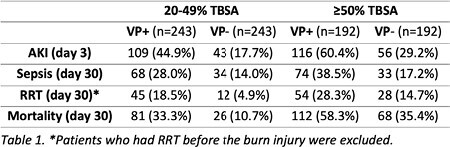# 703 Vasopressors in Early Resuscitation of Severe Burn Injuries

**DOI:** 10.1093/jbcr/irae036.248

**Published:** 2024-04-17

**Authors:** Kristine Knappskog, Juquan Song, Amina E I ayadi, Georgiy Golovko, Julia Kleinhapl, Anne Berit Guttormsen, Stian K Almeland, Steven E Wolf

**Affiliations:** University of Texas Medical Branch at Galveston and University of Bergen, Galveston, TX; University of Texas Medical Branch at Galveston, Galveston, TX; University of Texas Medical Branch, Galveston, TX; Department of Surgery, Division of Surgical Sciences, Medical Branch, University of Texas, Galveston, TX, USA; Division of Plastic, Aesthetic and Reconstructive Surgery, Department of Surgery, Medical University of Graz, Graz, Austria, Galveston, TX; University of Bergen, Bergen, Hordaland; University of Texas Medical Branch, Blocker Burn Unit, Galveston, TX; University of Texas Medical Branch at Galveston and University of Bergen, Galveston, TX; University of Texas Medical Branch at Galveston, Galveston, TX; University of Texas Medical Branch, Galveston, TX; Department of Surgery, Division of Surgical Sciences, Medical Branch, University of Texas, Galveston, TX, USA; Division of Plastic, Aesthetic and Reconstructive Surgery, Department of Surgery, Medical University of Graz, Graz, Austria, Galveston, TX; University of Bergen, Bergen, Hordaland; University of Texas Medical Branch, Blocker Burn Unit, Galveston, TX; University of Texas Medical Branch at Galveston and University of Bergen, Galveston, TX; University of Texas Medical Branch at Galveston, Galveston, TX; University of Texas Medical Branch, Galveston, TX; Department of Surgery, Division of Surgical Sciences, Medical Branch, University of Texas, Galveston, TX, USA; Division of Plastic, Aesthetic and Reconstructive Surgery, Department of Surgery, Medical University of Graz, Graz, Austria, Galveston, TX; University of Bergen, Bergen, Hordaland; University of Texas Medical Branch, Blocker Burn Unit, Galveston, TX; University of Texas Medical Branch at Galveston and University of Bergen, Galveston, TX; University of Texas Medical Branch at Galveston, Galveston, TX; University of Texas Medical Branch, Galveston, TX; Department of Surgery, Division of Surgical Sciences, Medical Branch, University of Texas, Galveston, TX, USA; Division of Plastic, Aesthetic and Reconstructive Surgery, Department of Surgery, Medical University of Graz, Graz, Austria, Galveston, TX; University of Bergen, Bergen, Hordaland; University of Texas Medical Branch, Blocker Burn Unit, Galveston, TX; University of Texas Medical Branch at Galveston and University of Bergen, Galveston, TX; University of Texas Medical Branch at Galveston, Galveston, TX; University of Texas Medical Branch, Galveston, TX; Department of Surgery, Division of Surgical Sciences, Medical Branch, University of Texas, Galveston, TX, USA; Division of Plastic, Aesthetic and Reconstructive Surgery, Department of Surgery, Medical University of Graz, Graz, Austria, Galveston, TX; University of Bergen, Bergen, Hordaland; University of Texas Medical Branch, Blocker Burn Unit, Galveston, TX; University of Texas Medical Branch at Galveston and University of Bergen, Galveston, TX; University of Texas Medical Branch at Galveston, Galveston, TX; University of Texas Medical Branch, Galveston, TX; Department of Surgery, Division of Surgical Sciences, Medical Branch, University of Texas, Galveston, TX, USA; Division of Plastic, Aesthetic and Reconstructive Surgery, Department of Surgery, Medical University of Graz, Graz, Austria, Galveston, TX; University of Bergen, Bergen, Hordaland; University of Texas Medical Branch, Blocker Burn Unit, Galveston, TX; University of Texas Medical Branch at Galveston and University of Bergen, Galveston, TX; University of Texas Medical Branch at Galveston, Galveston, TX; University of Texas Medical Branch, Galveston, TX; Department of Surgery, Division of Surgical Sciences, Medical Branch, University of Texas, Galveston, TX, USA; Division of Plastic, Aesthetic and Reconstructive Surgery, Department of Surgery, Medical University of Graz, Graz, Austria, Galveston, TX; University of Bergen, Bergen, Hordaland; University of Texas Medical Branch, Blocker Burn Unit, Galveston, TX; University of Texas Medical Branch at Galveston and University of Bergen, Galveston, TX; University of Texas Medical Branch at Galveston, Galveston, TX; University of Texas Medical Branch, Galveston, TX; Department of Surgery, Division of Surgical Sciences, Medical Branch, University of Texas, Galveston, TX, USA; Division of Plastic, Aesthetic and Reconstructive Surgery, Department of Surgery, Medical University of Graz, Graz, Austria, Galveston, TX; University of Bergen, Bergen, Hordaland; University of Texas Medical Branch, Blocker Burn Unit, Galveston, TX

## Abstract

**Introduction:**

Fluid resuscitation is the main treatment for preventing burn shock. Rescue therapies are usually initiated if patients respond poorly to fluid resuscitation with crystalloids alone. Vasopressors, often in combination with colloids, have been used to limit fluid needs. However, the effects of vasopressors during burn resuscitation remain inadequately investigated.

This study aims to identify the characteristics of patients receiving vasopressors during burn resuscitation and to establish potential correlations between vasopressor use and patient outcomes.

**Methods:**

A large patient database was searched for patients over ≥18 years of age with ≥20% total body surface area (% TBSA) burns. Two cohorts (VP+ and VP-) were established to assess the impact of vasopressors during the initial 24 hours after injury. The cohorts were then stratified by %TBSA. Propensity score matching was performed based on age, gender, ethnicity, race, and comorbidities. Statistical analysis was performed using two sample Z-test, risk ratio (RR) and odds ratio (OR), with p < 0.05 considered significant.

**Results:**

Following propensity matching, the VP+ cohort had a notably higher prevalence of burns exceeding 50% TBSA compared to the VP- cohort (45.9% vs. 27.8%, p < 0.05). Moreover, VP+ patients had a significantly increased incidence of inhalation injuries (67.9% vs. 38.5%, p < 0.05) and were more than twice as likely to require mechanical ventilation within the first day after burn injury (81.0% vs. 31.5%, p < 0.05). During the same period, 28.2% of VP+ patients and 13.0% of VP- patients underwent burn surgery (p < 0.05).

To account for burn severity differences, the cohorts were stratified by %TBSA into subgroups: 20-49% and ≥50%. Both VP+ subgroups had an increased risk of early acute kidney injury (AKI) and increased susceptibility to sepsis, greater need for renal replacement therapy (RRT), and elevated mortality rates by day 30 after burn injury (Table 1). Comparing the VP+ to the VP- cohort, the ORs and RRs for mortality were 4.17 (95% CI 2.57, 6.79) and 3.12 (95% CI 2.08, 4.67) in the 20-49% TBSA subgroup and 2.55 (95% CI 1.69, 3.86) and 1.65 (95% CI 1.32, 2.06) in the ≥50% TBSA subgroup. Similarly, for AKI development, an OR of 3.78 (95% CI 2.50, 5.73) and RR of 2.54 (95% CI 1.87, 3.44) was found in the 20-49% TBSA subgroup, and 3.71 (95% CI 2.42, 5.67) and 2.07 (95% CI 1.62, 2.66) in the ≥50% TBSA subgroup when comparing VP+ to VP-.

**Conclusions:**

After accounting for comorbidities, burn injury patients receiving vasopressors during fluid resuscitation had more severe injuries and faced increased risks of adverse outcomes, including AKI, sepsis, and mortality.

**Applicability of Research to Practice:**

This study raises the question of whether the adverse outcomes observed in burn patients receiving vasopressors are caused by the drugs themselves or merely reflect more severely ill patients. Understanding this distinction could help refine treatment strategies and ensure better outcomes for burn patients.